# Stillbirths in a large Shanghai maternity centre (2014–2024): trends, sex distribution, and gestational age-specific risk

**DOI:** 10.1186/s12884-026-08723-z

**Published:** 2026-02-06

**Authors:** Rui-Hong Xue, Ying Zhang, Dan Tang, Yun-Xia Li, Juan Li, Lin Zhang

**Affiliations:** 1 International Peace Maternity and Child Health Hospital, School of Medicine, Shanghai Jiao Tong University, No.910 Hengshan Road, Shanghai, 200030 China; 2 Shanghai Key Laboratory of Embryo Original Diseases, Shanghai , 200030 China; 3 Institute of Birth Defects and Rare Diseases, School of Medicine, Shanghai Jiao Tong University, Shanghai , 200030 China; 4 Karamay Central Hospital, Karamay, 834000 China

**Keywords:** Stillbirth, Prevalence, Sex ratio, Gestational age

## Abstract

**Background:**

Stillbirth is a catastrophic adverse pregnancy outcome that harms families and is a major global public health concern; however, the available data regarding its prevalence, sex disparities, and gestational age distribution (key for targeted interventions) are insufficient. This study addressed this gap in a large Shanghai pregnant cohort.

**Methods:**

This retrospective study included 140,594 pregnant women (after incomplete/nonsingleton cases were excluded) who delivered at a tertiary hospital (Oct 2014–Sep 2024). Stillbirth was defined per the WHO guidelines (≥ 28 weeks). Stillbirth rates were calculated overall and stratified by foetal sex/gestational age (28–31, 32–36, and ≥ 37weeks). Mann‒Kendall test was used to assess trends (2015–2023), chi-square tests were used to compare subgroups, and analyses were performed in R 4.5.1 (*P* < 0.05 indicates statistical significance).

**Results:**

Among 140,594 deliveries, 249 stillbirths occurred (rate: 0.18%; 95% CI: 0.16–0.20%). The increasing trend from 2015–2023 was nonsignificant (Z = 0.738, *P* = 0.461; tau = 0.229), with no sex disparities (males: 51.81%; 129/249; females: 48.19%; 120/249; χ²=0.325; *P* = 0.5684). Regardless of sex, stillbirths were more common at 28–31 weeks and fewer at ≥ 37 weeks (male: χ²=28.79; female: χ²=21.05; *P* < 0.001).

**Conclusions:**

Stillbirth rate in our maternity centre has remained consistently low over the past decade, with no significant temporal increase or sex-based disparities observed. The risk of stillbirth decreased with advancing gestational age, with the highest risk noted at 28–31 weeks; these findings underscore the need for targeted monitoring and interventions during the early phase of late pregnancy. Future multicentre studies are warranted to validate these findings and provide evidence to inform stillbirth prevention strategies.

**Supplementary Information:**

The online version contains supplementary material available at 10.1186/s12884-026-08723-z.

## Background

Stillbirth, defined as foetal death occurring after 28 weeks of pregnancy by the World Health Organization (WHO), has deep and long-lasting effects on parents, healthcare providers, and society and is a significant public health problem worldwide [1–3]. In 2021, the global stillbirth rate was 23.0 per 1000 births at 20 weeks of gestation or longer, compared to 16.1 per 1000 births at 28 weeks of gestation or longer [4]. The stillbirth rate is a sensitive indicator of the quality of care during pregnancy and childbirth and a marker of health system strength [5]. Nevertheless, the number of stillborn infants might be underestimated, as stillbirths are often underreported [6, 7].

The stillbirth prevalence at the community level is typically less than 1% in more developed parts of the world and can exceed 3% in less developed regions [8]. The United Nations’ Global Strategy urges that stillbirths be prioritized [9]. The Every Newborn Action Plan endorsed by the World Health Assembly in 2014 set a target of reducing the stillbirth rate (SBR) to < 12/1000 total births by 2030 [10]. However, reductions in stillbirths globally have been slow and are not on track to reach this target. To date, comprehensive epidemiological data on its prevalence and risk factors are lacking, which is essential for designing tailored interventions.

In China, a multicentre cross-sectional study of ninety‐six hospitals distributed across 24 (of 34) provinces reported that the incidence of stillbirth was 13.2 per 1000 births in 2015–2016 and that approximately one‐third of all stillbirths may be preventable [11]. Another retrospective study in Zhejiang Province, southern China, reported that the total incidence of third-trimester stillborn infants was 3.06/1000 (341/111,275) [12]. A population-based retrospective cohort of all births estimated an overall stillbirth rate of 4.5 per 1000 births among a total of 492,184 births, and migration might be a risk factor [13].

Different gestational ages and foetal sexes of stillborn infants might also play important roles in the pathogenesis of stillborn infants, but research on these factors is very limited. The International Classification of Diseases-10 (ICD-10) lacks a classification of stillbirths according to gestational age [14]. Moreover, abnormal placental perfusion (APP) is associated with and accounts for most of the unexplained stillbirth risk, and different mechanisms exist based on foetal sex [15]. In addition, in recent years, the world has been affected by COVID-19, which has already had a very large impact on healthcare. However, much remains to be learned about the relationship between COVID-19 and stillbirth. To date, evidence of the direct impact of COVID-19 on pregnancy outcomes is limited [16, 17].

In this study, we sought to investigate the prevalence of stillbirths and identify possible risk factors, including the distribution of gestational age and foetal sex, and analysed the correlation between the COVID-19 outbreak and stillbirth. This information might facilitate evidence-based clinical decision-making in high-risk pregnancies and enhance our understanding of stillbirths.

## Methods

### Study design and data source

After obtaining ethical approval from the local committee, we used data from the digital birth registration system of International Peace Maternity and Child Health Hospital, a large tertiary maternity centre in Shanghai, China, with approximately 10,000–15,000 annual low- and high-risk deliveries. The study included singleton deliveries from October 2014 to September 2024. At our centre, antepartum stillbirths predominate, with intrapartum stillbirths being extremely rare and classified as medical malpractice. The system routinely registers all ≥ 28-week deliveries (vaginal/caesarean section), capturing maternal age, gravidity, parity, gestational week, birth time, haemorrhage, 1′/5′ Apgar scores, newborn sex/weight, and hospital campus; midwives/surgical assistants complete registration post vaginal/caesarean section delivery. Notably, it is not a medical records system: patient diagnoses are incomplete, with only specific severe comorbidities recorded, precluding analysis of stillbirth correlations with most complications and comorbidities. However, core demographic and delivery-related data are complete and reliable, enabling calculation of stillbirth incidence and its associations with key clinical characteristics. A suburban campus (Fengxian District) opened in September 2022, alongside the urban Xuhui District campus, with unified medical staff and management; therefore we analysed stillbirth risk by hospital campus. Multiple gestations were excluded. Intrapartum stillbirths are exceptionally rare at our centre, as standardised obstetric care is implemented for prevention; such cases trigger clinical governance reviews, potential institutional indemnity payments, and regulatory inquiries, indicating that our dataset is dominated by antenatal foetal deaths unrelated to intrapartum management. While some cases may involve ≥ 28 week of pregnancy termination for foetal anomalies, our retrospective study, which relies on postdelivery Apgar scores as the primary data source, highlights the need for future research to adopt refined stillbirth classification systems based on pathological aetiology.

### Outcome variable and its selected indicators

The World Health Organization (WHO) guidelines define stillbirth as death prior to the complete expulsion or extraction from its mother of a product of human conception, where the foetus shows no signs of life—such as breathing, heartbeat, pulsation of the umbilical cord, or definite movement of voluntary muscles—irrespective of the duration of pregnancy [6]. In our study, stillbirth was defined as a birth with no signs of life, such as a heartbeat or a nonzero Apgar score, occurring at or after 28 weeks of gestation [18]. Stillbirths < 28 gestational age were excluded. Additionally, multiple gestations were excluded. Extreme gravidity (> 15) and parity (> 15) were excluded. Patients with exceptional error parity and missing information were also excluded.

### Statistical analysis

Descriptive statistics were used to profile the sample using ANOVA for continuous variables. To evaluate the associations between different characteristics and stillbirths, binary logistic regressions were performed. Odds ratios (95% CIs) were calculated for the maternal age ≥ 35-year-old group, parous group, suburban campus group, and post-COVID-19 period, with each group compared against its respective reference group. Statistical significance was set at *P* < 0.05. The analyses were conducted using SPSS 23 statistical software.

In the logistic regression model, we further incorporated maternal age as a continuous variable and added an additional “age-squared” term to test for potential U-shaped or J-shaped nonlinear relationships between age and stillbirth risk. The Mann‒Kendall (MK) test combined with Sen’s slope estimator was used to analyse annual trends in overall and sex-specific stillbirth rates: the MK test assesses trend presence and statistical significance by calculating sign changes between sequential data points, whereas Sen’s slope quantifies trend magnitude. The analyses covered the years 2015–2023 (2014 and 2024 were excluded because of incomplete annual data); key indicators were interpreted as follows: a positive Z score denotes an upwards trend (absolute values reflect trend strength), the P value (significance threshold *P* < 0.05) indicates the probability of observed trends arising from random chance, and Kendall’s tau coefficient (−1 to 1) is used to measure trend strength and direction, with |Tau|>0.6 (strong), 0.3<|Tau|<0.6 (moderate) and |Tau|<0.3 (weak) trends (positive values confirm upwards trends). The analyses were performed using R 4.5.1 statistical software.

## Results

**Fig. 1:**
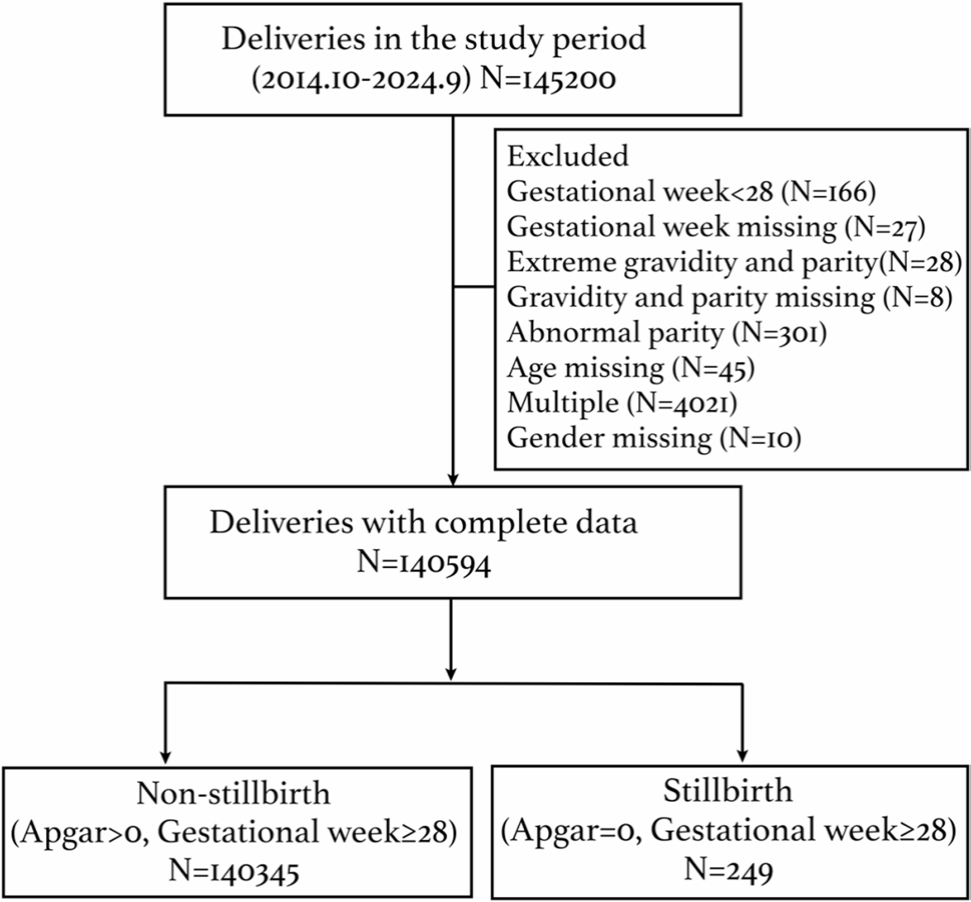
Flowchart for selecting the women included in this study

By retrospectively studying the patients’ digital records between October 2014 and September 2024, 145200 women who delivered at our hospital were included. Women whose gestational week was < 28 or whose gestational week was missing were excluded. Women with extreme gravidity or parity or those with abnormal or missing data were excluded. Women with missing ages or missing foetal sex data were excluded. Women with multiple gestations were excluded. Ultimately, 140594 deliveries with complete data were included in our final analyses, including 4207 deliveries in 2014 (October to December), 14389 deliveries in 2015, 16214 deliveries in 2016, 16523 deliveries in 2017, 15294 deliveries in 2018, 15411 deliveries in 2019, 13158 deliveries in 2020, 11964 deliveries in 2021, and 11094 deliveries in 2022 and 12108 deliveries in 2023 and 10232 deliveries in 2024 (January to September). Among 140594 deliveries, 249 were stillborn (5’ Apgar score = 0) and 140,345 were nonstillborn (5’ Apgar score > 0) (Fig. 1). The overall stillbirth rate was 1.8 per 1000 births (249/140594). (Table 1)

**Table 1:** Clinical characteristics of livebirths and stillbirths in the study period

Total deliveries	Nonstillbirth	(%)	Stillbirth	(%)
*N* = 140,594	*N* = 140,345	(99.82%)	*N* = 249	(0.18%)
Characteristics	Median (25th-75th)		Median (25th-75th)	
Maternal age (years)	31 (29, 34)		31 (28, 35)	
Gestational week	39.1 (38.3, 40)		31.5 (29.3, 35.1)	
Gravidity	2 (1, 2)		2 (1, 3)	
Parity	1 (1, 2)		1 (1, 2)	

The clinical characteristics of the women in the study are shown in Table 1. Among the 140,594 women included in the final analyses, the median (25th–75th percentile) maternal age was 31 (29–34) years, the number of gestational weeks was 39.1 (38.3, 40) weeks, the gravidity was 2 (1, 2) times, and the parity was 1 (1, 2) time. Among the 249 women with stillbirths, the median (25th–75th percentile) maternal age was 31 (28–35) years, the number of gestational weeks was 31.5 (29.3, 35.1) weeks, the gravidity was 2 (1, 3) times, and the parity was 1 (1, 2) time

**Fig. 2:**
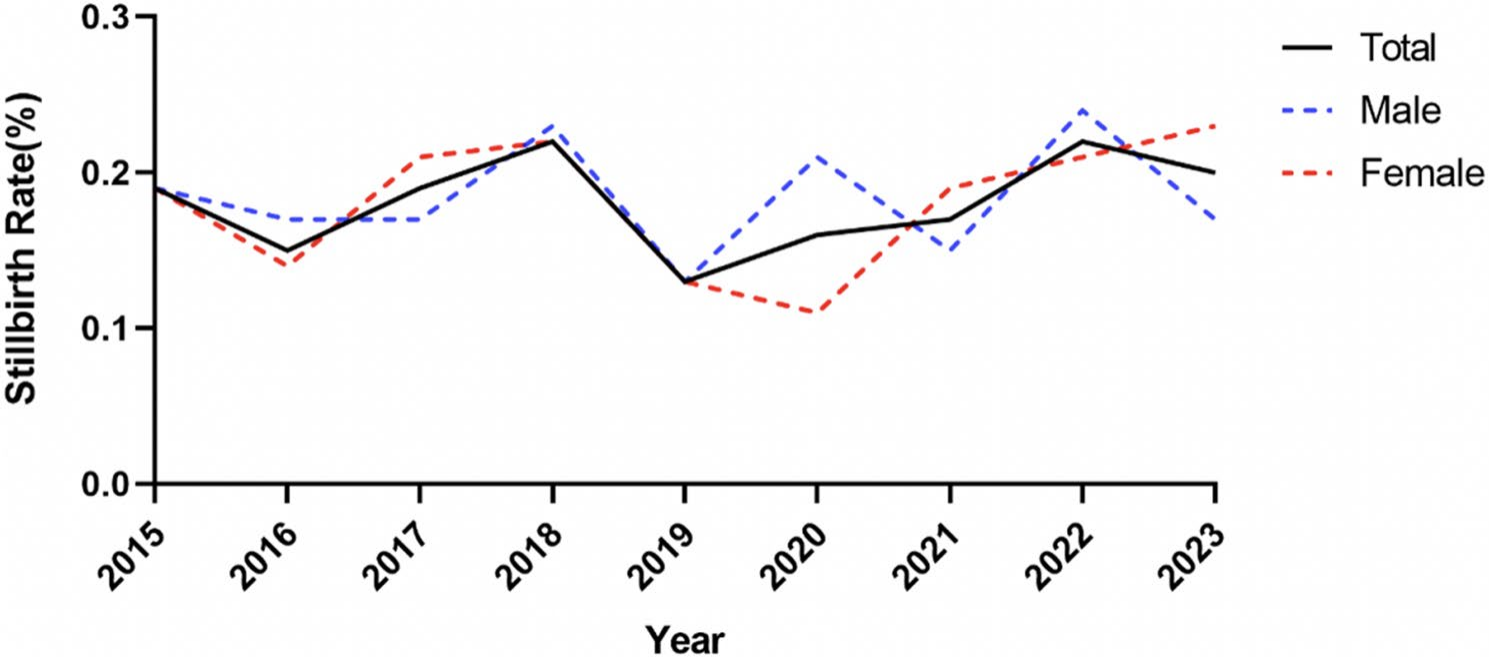
Alterations in stillbirth rates during the study period. The alterations in stillbirth rates from 2015 to 2023, including the total stillbirth rates and among different newborn sexes, are shown in Fig. 2. The positive value (Z=0.738) indicates an upwards trend in the stillbirth rate from 2015 to 2023, while the P value is 0.461 and Tau=0.229 (Kendall's Tau coefficient), which indicates that the observed upwards trend over the past 9 years is not statistically significant and that this change is likely caused by random fluctuations rather than a definite, monotonic upwards trend. The Sen’s slope for females is 0.004, that for males is 0, and that for the entire sample is 0.003. (Mann‒Kendall trend test)

Since the data from both 2014 (October onwards) and 2024 (before September) did not span one full year, we excluded these data when we examined the overall stillbirth trend. Thus, the incidence rates of stillbirth were calculated per year from 2015 to 2023. The stillbirth rates were 0.19% in 2015, 0.15% in 2016, 0.19% in 2017, 0.22% in 2018, 0.13% in 2019, 0.16% in 2020, 0.17% in 2021, 0.23% in 2022, and 0.20% in 2023. We used the Mann‒Kendall trend test to assess the trend in stillbirth rates over the past years. A positive value (Z = 0.738) indicates an upwards trend in the stillbirth rate from 2015 to 2023, with *P* = 0.461 and Tau = 0.229 (Kendall’s Tau coefficient). These findings indicate that the observed upwards trend over the past years is not statistically significant, and that this change is likely caused by random fluctuations rather than a definite, monotonic upwards trend (Fig. 2).

Among 249 stillborn cases, 129 were male, and 120 were female, with stillbirth rates of 51.81% (129/249) for males and 48.19% (120/249) for females. (χ² = 0.325, *p* = 0.5684)

Table 2 shows the clinical characteristics and sex distributions of stillborn infants in different gestational week subgroups. In this table, stillbirths were divided according to the number of gestational weeks. In the 28–31-week subgroup, 70 male and 60 female stillborn infants were included. In the 32–36-week subgroup, 38 male and 41 female stillborn infants were included. In the ≥ 37-week subgroup, there were 21 male and 19 female stillbirths. No statistically significant difference in the stillbirth rate was noted based on sex. (χ²=0.659, *P* = 0.719, Pearson’s chi-square test; Table 2 and Figure S1)

**Table 2:** Clinical characteristics and gender distributions of stillbirths in different gestational week subgroups

Total (N=249)	28-31weeks		32-36 weeks		>=37 weeks	
	Stillbirths,130 (52.21%)		Stillbirths,79 (31.73%)		Stillbirths,40 (16.06%)	
Characteristics	Male, 70	Female, 60	Male, 38	Female, 41	Male, 21	Female, 19
	53.85%	46.15%	48.10%	51.90%	52.50%	47.50%
	Median (25th −75th)		Median (25th −75th)		Median (25th −75th)	
Maternal age(y)	30 (28, 34)		32 (29, 36)		32 (30, 36)	
Gestational week(w)	29.3 (28.5, 30.4)		34 (32.6, 35.3)		38.75 (37.6, 39.5)	
Gravidity(n)	2 (1, 3)		2 (1, 3)		2(1, 3)	
Parity(n)	1 (1, 2)		1 (1, 2)		1(1, 2)	

Table 2 shows the clinical characteristics and sex distributions of stillborn infants in different gestational week subgroups. There were 249 women with stillborn infants, among whom 130 (52.21%) were stillborn at 28–31 gestational weeks, 79 (31.73%) were stillborn at 32–36 gestational weeks, and 40 (16.06%) were stillborn at ≥37 gestational weeks

**Fig. 3:**
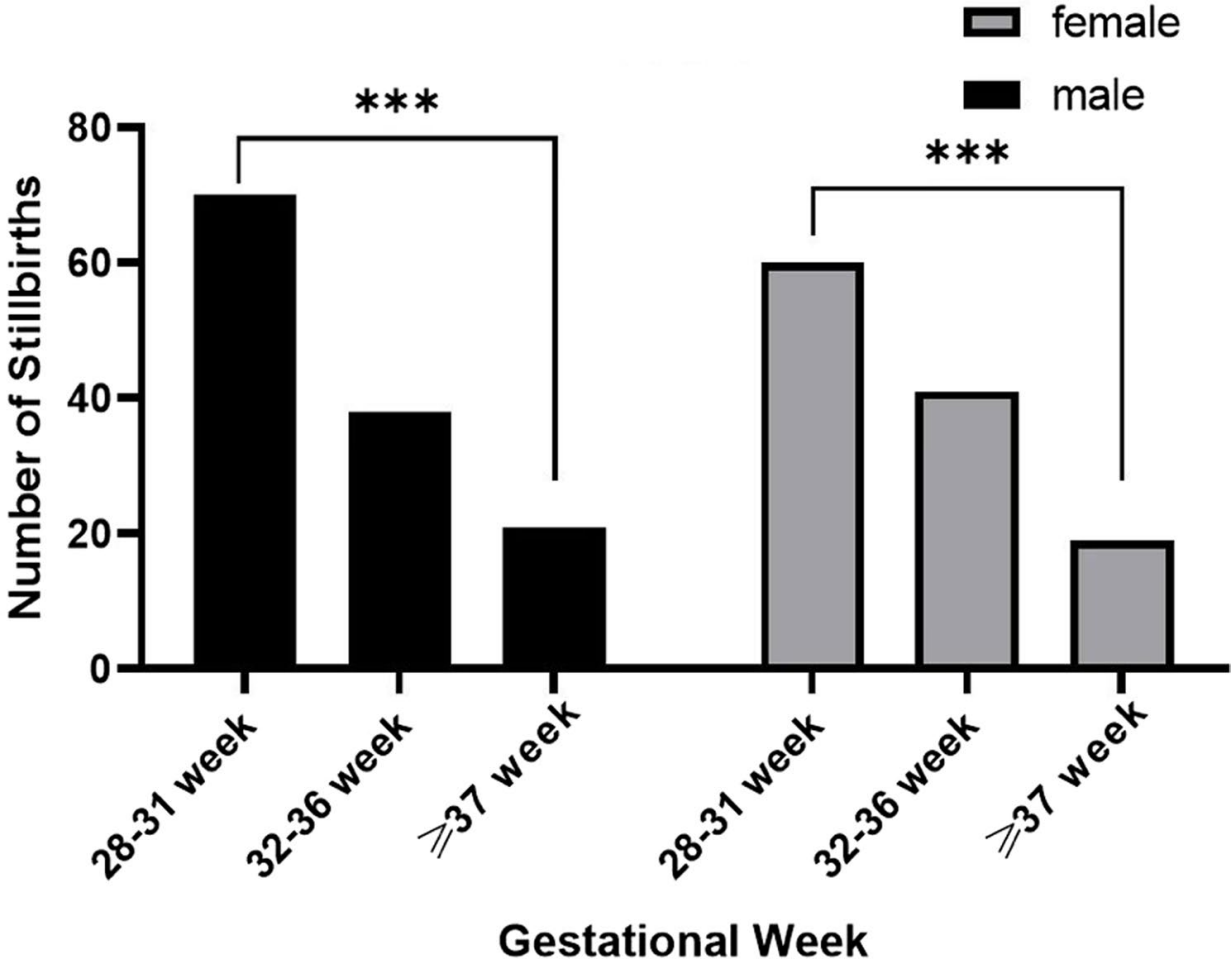
Sex distributions of stillborn infants in different gestational age subgroups. Compared with the hypothesis of “equal number of stillbirths in the three groups”, regardless of sex, there were significantly more stillbirths at 28–31 weeks and significantly fewer stillbirths after 37 weeks of gestation (χ²=28.79 (male) and 21.05 (female); *p*<0.001) (chi-square goodness-of-fit test)

The sex distributions of stillborn infants across different gestational age subgroups are shown in Fig. 3. Compared with the hypothesis of “equal number of stillbirths in the three groups”, regardless of sex, there were significantly more stillbirths at 28–31 weeks of gestation and significantly fewer stillbirths after 37 weeks of gestation (χ²=28.79 (male) and 21.05 (female), *p* < 0.001; chi-square goodness-of-fit test; Fig. 3).

**Fig. 4:**
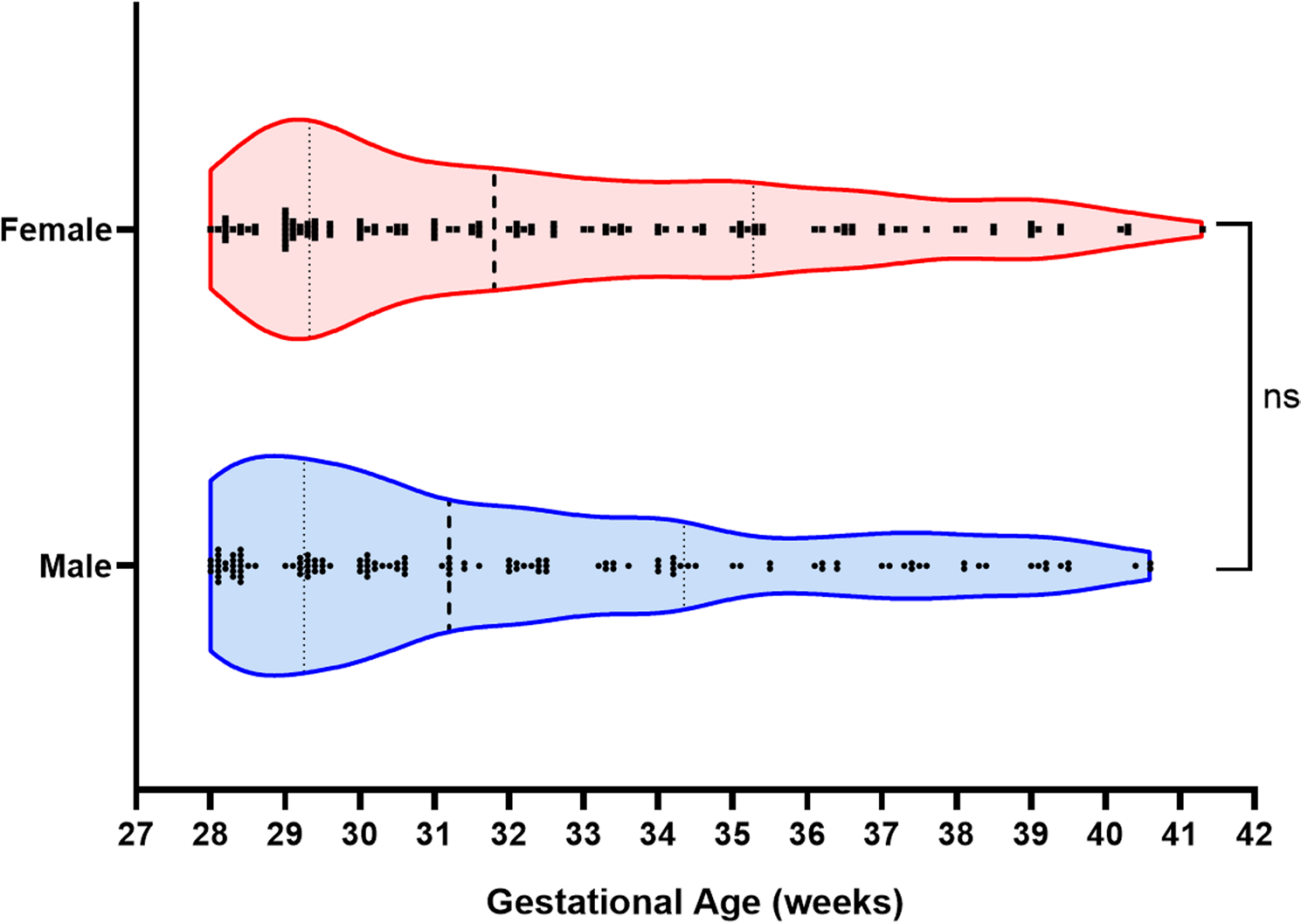
Distribution of stillbirths among different sexes by gestational age. The Violin plot reveals the gestational age distribution of stillborn infants by sex. Because the “gestational age” data did not follow a normal distribution, a nonparametric Mann‒Whitney test was chosen. The results revealed no significant difference between the gestational age distributions of male and female stillborn children (P=0.2835, Mann‒Whitney U test). These findings indicate that the gestational age distribution of stillborn infants is similar across the different genders. The risk of stillbirth is not significantly different for premature birth, full-term birth, or postterm birth

The distribution of stillbirths by sex across different gestational ages is shown in Fig. 4. The Violin Plot reveals the gestational age distribution of stillborn infants according to sex. Because the “gestational age” data did not follow a normal distribution, a nonparametric Mann‒Whitney U test was employed. The results revealed no significant difference between the gestational age distributions of male and female stillborn children (*P* = 0.2835, Mann‒Whitney U test). These findings indicate that the gestational age distribution of stillborn infants is similar across the different genders. The risk of stillbirth is not significantly different for premature birth, full-term birth, or postterm birth. (Fig. 4)

Table 3 shows the logistic regression analysis of the correlation between stillbirth occurrence and different characteristics. Among the 140,594 deliveries, 249 were stillborn, and the stillbirth incidence rate was 0.18%. We further analysed the correlation between the occurrence of stillbirth and clinical characteristics, including maternal age, parity, different hospital campuses and the COVID-19 outbreak. We found that there were no statistically significant differences between the risk of stillbirth and maternal age [odds ratio (95% CI), 1.23 (0.93, 1.63)], parity [OR (95% CI), 0.98 (0.74, 1.29)], hospital campuses [OR (95% CI), 0.73 (0.42, 1.25)], or the COVID-19 outbreak [OR (95% CI), 1.06 (0.82, 1.36)], with *P* > 0.05. (Table 3)

**Table 3:** Logistic analysis of the correlation between stillbirth occurrence and different variables

Characteristics	Total Deliveries		Stillbirths			
	Deliveries, *n*†	(%)	Stillbirths, *n*	(%)	Odds ratio (95% CI)	*P* value
Total	140,594	(100)	249	(0.18)		
Maternal age(years)						
< 35 (reference)	109,216	(77.68)	184	(0.17)	1 (reference)	
≥ 35	31,378	(22.32)	65	(0.21)	1.23 (0.93, 1.63)	0.152
Parity (n)						
Nulliparous (reference)	99,846	(71.02)	178	(0.18)	1 (reference)	
Not nulliparous	40,748	(28.98)	71	(0.17)	0.98 (0.74, 1.29)	0.870
Birthplace						
Residents (reference)	87,963	(62.57)	125	(0.14)	1 (reference)	
Immigrants	26,731	(19.01)	45	(0.17)	1.19 (0.84, 1.67)	0.330
Missing	25,900	(18.42)	79	(0.31)		
Delivery Hospital Campus						
Urban (reference)	129,956	(92.43)	235	(0.18)	1 (reference)	
Suburban	10,638	(7.57)	14	(0.13)	0.73 (0.42, 1.25)	0.250
COVID-19 outbreak ^**θ**^						
Before COVID-19	82,039	(58.35)	142	(0.17)	1 (reference)	
After COVID-19	58,555	(41.65)	107	(0.18)	1.06 (0.82, 1.36)	0.670

^θ^COVID-19 outbreak time divide was determined at January 1 st, 2020 in the analyses

†This sum does not necessarily equal the sample size for all variables because some data were missing

Table 3 shows the logistic regression analysis of the correlation between stillbirth occurrence and different variables. Among the 140594 deliveries, 249 were stillborn, and the stillbirth incidence rate was 0.18%. We further analysed the correlation between the occurrence of stillbirth and clinical characteristics, including maternal age, parity, different hospital campuses and the COVID-19 outbreak. No statistically significant differences were noted between the risk of stillbirth and maternal age [odds ratio (95% CI), 1.23 (0.93, 1.63)], parity [OR (95% CI), 0.98 (0.74, 1.29)], hospital campuses [OR (95% CI), 0.73 (0.42, 1.25)], and the COVID-19 outbreak [OR (95% CI), 1.06 (0.82, 1.36)], with *P*>0.05

Although male stillborn patients outnumbered female stillborn patients between 28 and 31 weeks of gestation, a slight predominance of female cases was observed at 32–36 weeks (Fig. S1). However, at full term, males again became marginally more prevalent. Statistical analysis revealed no significant differences in the proportional distribution across the three gestational age groups (χ²=0.659, *P* = 0.719, Pearson’s chi-square test; Fig. S1).

 The U-shaped association between maternal age and stillbirth risk (quadratic term *P* = 5.71 × 10⁻⁶) is shown in Fig. S2, with the lowest risk observed at 32.9 years; notably, women ≤ 20 years old had a 3.54-fold greater risk than this optimal age, exceeding the risk elevation seen in 40-year-old women (1.46-fold) (Figure S2).

## Discussion

In this retrospective study, the results revealed a progressive decline in stillbirth rates with advancing gestational age during late pregnancy, despite no significant increase in stillbirth rates over the past decade and no sex-based disparity in stillbirth incidence.

Globally, some progress has been made in reducing the stillbirth rate over the past two decades, with a reduction of 35%, from 21.4 stillbirths per 1,000 total births in 2000 to 13.9 in 2019. In addition, the total number of stillbirths declined by 32%, from 2.9 million in 2000 to 2.0 million in 2019. However, these declines have not kept pace with the progress in under-five mortality. Stillbirths in the Volta region of Ghana have declined in recent years [19], while, whereas in many sub-Saharan African countries, the number of stillborn cases has stagnated or has even increased, as population growth has outpaced decreases in the stillbirth rate [20].

Why is progress in reducing the stillbirth rate so slow? Some reasons might include the absence of or poor quality of care during pregnancy and birth, a lack of investment, inadequate social recognition of stillbirths as a burden on families, measurement challenges and major data gaps, the absence of leadership, and the absence of established sustainable global targets [20]. There are many challenges in the global research of stillborn infants because of incomplete data. Strengthening stillbirth registration and utilizing advanced spatial methods are crucial for improved monitoring and intervention planning [21]. The UN Interagency Group for Child Mortality Estimation (UN IGME), together with its Technical Advisory Group and Core Stillbirth Estimation Group, has developed robust methods to estimate stillbirths [22]. Precise, high-quality and complete data on stillborn children are important for promoting better health and social policies and protecting families. In our study, the advanced information operation and delivery registration system allowed us to acquire important delivery data for further analyses. To prevent stillbirths, we need to provide data and evidence to answer various questions [20].

Different causes might account for the occurrence of stillbirth. Obstetric complications, placental diseases, genetic anomalies, and infections could be reasons for stillbirths, and some cases were considered idiopathic. In low-income countries, stillbirths are often attributed to infection and complications during labour and birth. However, in middle- and high-income countries, stillbirths are reported as placental complications [23]. The Stillbirth Collaborative Research Network Writing Group reported that obstetric conditions and placental abnormalities were the most common causes of stillbirth, although the distribution differed by race/ethnicity [24]. In a retrospective analysis of 566 stillborn cases, Leila Caillault and colleagues reported that placental vascular anomalies were the most frequent cause of stillbirth (36%), followed by umbilical cord complications (11%) and infections (9%), and stillbirth remained unexplained in 17% of the cases [25]. Evidence for the familial aggregation of stillborn infants has been reported and warrants investigation into genes associated with stillborn infants [26].

In this study, we report a consistently low and stable stillbirth rate of 1.8 per 1000 deliveries over the past decade at this Shanghai maternity centre. This rate (1.8‰) remains at a globally low level and compares favourably with the international average of 16.1‰ [4]. This merit is inextricably linked to the dedicated efforts of medical staff and administrators, as well as the municipal government’s sustained investment in high-risk maternal care over the years. The maternal risk stratification system (colour-coded as green, yellow, orange, purple, and red) enables early pregnancy classification of antenatal patients on the basis of comorbidities, with real-time dynamic adjustments. Maternal education programmes provide integrated perinatal guidance: foetal heart rate monitoring begins at 32 weeks for high-risk pregnancies and 34 weeks for low-risk pregnancies. Abnormal readings prompt reassessment, with hospital admission for severe cases. High-risk late-pregnancy patients may borrow remote foetal monitors for clinicians to review home telemonitoring data. Rare late-pregnancy stillbirths often spark aggression, mostly verbal abuse of staff, creating major management challenges and significant psychological distress for healthcare professionals. Thus, strengthening prenatal and antenatal healthcare management is important for reducing the risk of stillbirth, improving pregnancy outcomes, and promoting maternal and child health [27].

Foetal sex influences pregnancy and its outcomes due to hormonal and chromosomal differences [28],and the sex ratio of stillbirths varies considerably across studies. In our study, we found no significant geospatial disparity in sex-specific stillbirth rates, consistent with the reported literature from other regions of the world [21]. However, a small number of reports have suggested a higher risk of stillbirth in male foetuses [29–31], but some have suggested that stillbirth is higher among females than among males [32]. We stratified stillborn children into three gestational age subgroups in our study, and no statistically significant differences in sex distribution were noted across these subgroups.

Crucially, stillbirths at varying gestational ages may involve different mechanisms. Multicountry analyses of births revealed that almost two-thirds to three-quarters of stillbirths were born preterm and that one-fifth were small-for-gestational age (SGA), with the highest stillbirth rates associated with the coexistence of preterm birth and SGA. These studies suggest the need for further analyses to better understand the patterns of gestation-specific risk in these populations [33, 34]. However, these two studies merely examined the co-occurrence and did not establish a causal relationship between the two. Consequently, a more detailed investigation into the gestational age distribution of stillborn infants may provide valuable insights into the underlying causes, thereby facilitating the identification of contributory factors. Notably, distinct placental lesions are present across different gestational ages [35], and distinguishing stillborn infants at different gestational ages is associated with specific causes of death. In extreme preterm stillbirth (PTSB) (< 28 weeks), obstetric complications and infections are associated with acute funisitis. In early PTSB (28–33+6/7 weeks), uteroplacental insufficiency was associated with parenchymal infarcts. In term stillborn patients (≥ 37 weeks), increased syncytial knots were associated with umbilical cord causes and infection [36].

Notably, the impact of COVID-19 on stillbirth rates depends on the complex interactions among socioeconomic status, healthcare access, infection status and gestational timing of exposure. Despite fears that pandemic-related healthcare restrictions, financial hardship and higher stress would worsen pregnancy outcomes, stillbirth rates remained stable [37], and no significant differences in the stillbirth populations were noted between the prepandemic and pandemic periods [38]. Stillbirth remains an ongoing global concern. Evidence suggests that the rate has increased during the COVID-19 pandemic, but mostly in low- and middle-income countries, and strict access to healthcare during the pandemic is potentially a major factor [39]. Another study revealed that COVID-19 was linked to an elevated stillbirth risk only when pregnant people were infected during early-to-mid pregnancy, not during the third trimester or near delivery, suggesting that the foetus may be particularly vulnerable to SARS-CoV-2 during early pregnancy [40]. In our study, the assessment of the impact of COVID-19 should be interpreted with caution, particularly given the lack of infection status data, healthcare access indicators, and stratified analyses.

The concentrated stillbirth risk at 28–31 weeks observed in our cohort highlights the urgent need for gestation-specific clinical interventions. Emerging evidence links early preterm stillbirths (28–33⁺^6^/_7_ weeks) to uteroplacental insufficiency and parenchymal infarcts, which are pathologies that may be detectable via enhanced antenatal surveillance. We therefore propose three targeted measures: first, integrating Doppler ultrasound assessment of uterine artery blood flow into routine antenatal care for high-risk pregnancies starting at 26 weeks to identify placental dysfunction early; second, training midwives and obstetricians in recognizing subtle signs of foetal compromise (e.g., reduced foetal movement) in this gestational window, alongside patient education on symptom reporting; and third, establishing a dedicated preterm stillbirth review team to analyse cases at 28–31 weeks and identifying modifiable factors (e.g., suboptimal monitoring frequency) for quality improvement.

While our single-centre data from a large tertiary Shanghai maternity centre provide robust insights into urban Chinese stillbirth patterns, generalisability to rural and other urban Chinese settings is limited—particularly due to regional disparities in healthcare access and maternal sociodemographic characteristics. Future multicentre research across diverse geographic regions (e.g., Shanghai vs. inland provinces) should validate our findings on gestational age risk gradients and sex neutrality. Integrating placental histopathology and maternal comorbidity data (e.g., gestational diabetes and hypertensive disorders), both of which are lacking in our dataset, into such designs could also clarify the aetiological drivers of 28–31-week stillbirths, addressing a key gap in our analysis. Moreover, recent research has shown that aspirin may prolong the gestational age at delivery (GAD) in patients with placental dysfunction (PD), with delays of 2.09 and 1.33 weeks observed at 28 and 32 weeks of gestation, respectively [41]. Such efforts would strengthen the evidence base for national stillbirth prevention strategies, a key priority for both clinical practice and public health in China.

Our study has key strengths: a large sample size, standardised healthcare provision and a homogeneous pregnant population. Complete clinical data allowed us to analyse changes in stillbirth rates; plot trend curves; examine sex ratios and gestational ages; and further explore confounding factors such as maternal age, parity and birthplace. Several limitations also need to be noted. First, this was a single-centre, hospital-based study; ethnic homogeneity increased internal validity but reduced the generalisability of our findings. Second, incomplete diagnostic data prevented further analysis of the links between stillbirth and pregnancy comorbidities in the large cohort. Key prenatal markers, including birth weight and foetal growth status, were not included, limiting in-depth investigations of the potential pathophysiological mechanisms underlying some stillbirths. Third, incomplete diagnostic records in the birth registration system meant that we could not further analyse or classify stillbirth causes, such as identifying those from pregnancy termination due to foetal malformation separately. Stratified, in-depth investigation of stillbirth cases, by gestational age and aetiology, would be highly valuable. It would deepen our understanding of stillbirth pathogenesis and help shape clinical prevention strategies to validate these findings.

## Conclusion

Stillbirth rate in our maternity centre has remained consistently low over the past decade, with no significant temporal increase or sex-based disparities observed. The risk of stillbirth decreased with advancing gestational age, with the highest risk noted at 28–31 weeks; these findings underscore the need for targeted monitoring and interventions during the early phase of late pregnancy. Future multicentre studies are warranted to validate these findings and provide evidence to inform stillbirth prevention strategies.

## Supplementary Information

Supplementary Material 1: Fig. S1 Comparison of stillborn sex by gestational age subgroup. Although male stillborn patients outnumbered female stillborn patients between 28 and 31 weeks of gestation, a slight predominance of female cases was observed at 32–36 weeks (Table S1). However, at full term, males again became marginally more prevalent. Statistical analysis revealed no significant differences in the proportional distribution across the three gestational age groups (χ²=0.659, P=0.719; Pearson’s chi-square test). Fig. S2 Predicted probability curve of stillbirth risk by maternal age. Using maternal age as a continuous variable with an added quadratic term in logistic regression, this plot illustrates the nonlinear U-shaped relationship between maternal age and stillbirth risk; the trough of the curve corresponds to 32.9 years, and young women (≤20 years) showed a more pronounced increase in risk than advanced-age women (40 years).

## Data Availability

The data generated and/or analysed during the current study are available from the corresponding author upon reasonable request.

## Abbreviations

APP: Abnormal Placental Perfusion
COVID-19: Coronavirus Disease 2019
DCDA: Dichorionic Diamniotic
ICD-10: International Classification of Diseases, 10th Revision
MCDA: Monochorionic Diamniotic
PD: Placental Dysfunction
PTSB: Preterm Stillbirth
SBR: Stillbirth Rate
SGA: Small-for-Gestational Age
UN IGME: United Nations Interagency Group for Child Mortality Estimation
