# Supplementary material for: Stillbirths in a large Shanghai maternity centre (2014–2024): trends, sex distribution, and gestational age-specific risk
Source: BMC Pregnancy Childbirth. 2026 Feb 6;26:312. doi: 10.1186/s12884-026-08723-z (PMC13011506; doi:10.1186/s12884-026-08723-z)
Supplement: Supplementary file 1 — Supplementary Material 1: Fig. S1 Comparison of stillborn sex by gestational age subgroup. Although male stillborn patients outnumbered female stillborn patients between 28 and 31 weeks of gestation, a slight predominance of female cases was observed at 32–36 weeks (Table S1). However, at full term, males again became marginally more prevalent. Statistical analysis revealed no significant differences in the proportional distribution across the three gestational age groups (χ²=0.659, P=0.719; Pearson’s chi-square test). Fig. S2 Predicted probability curve of stillbirth risk by maternal age. Using maternal age as a continuous variable with an added quadratic term in logistic regression, this plot illustrates the nonlinear U-shaped relationship between maternal age and stillbirth risk; the trough of the curve corresponds to 32.9 years, and young women (≤20 years) showed a more pronounced increase in risk than advanced-age women (40 years). [file 12884_2026_8723_MOESM1_ESM.docx]

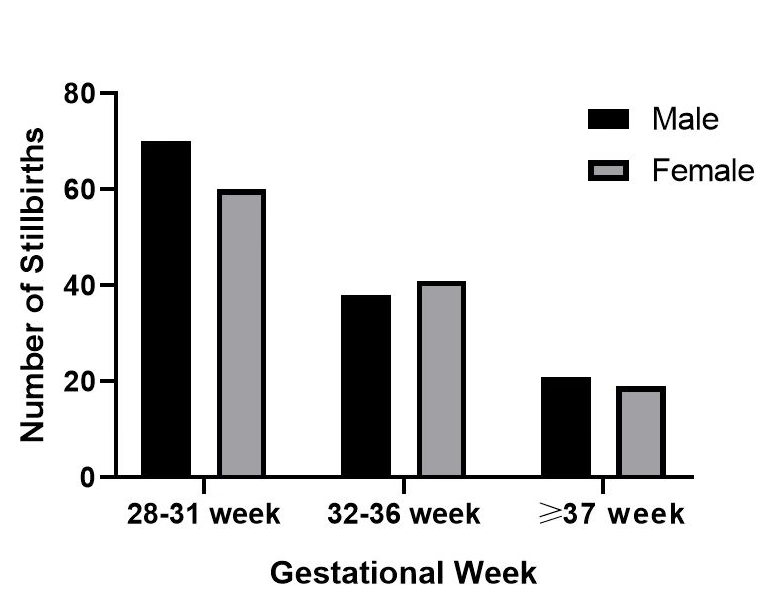


**Fig. S1 Comparison of stillborn sex by gestational age subgroup**.

Although male stillborn patients outnumbered female stillborn patients between 28 and 31 weeks of gestation, a slight predominance of female cases was observed at 32–36 weeks (Table S1). However, at full term, males again became marginally more prevalent. Statistical analysis revealed no significant differences in the proportional distribution across the three gestational age groups (χ²=0.659, P=0.719; Pearson’s chi-square test).


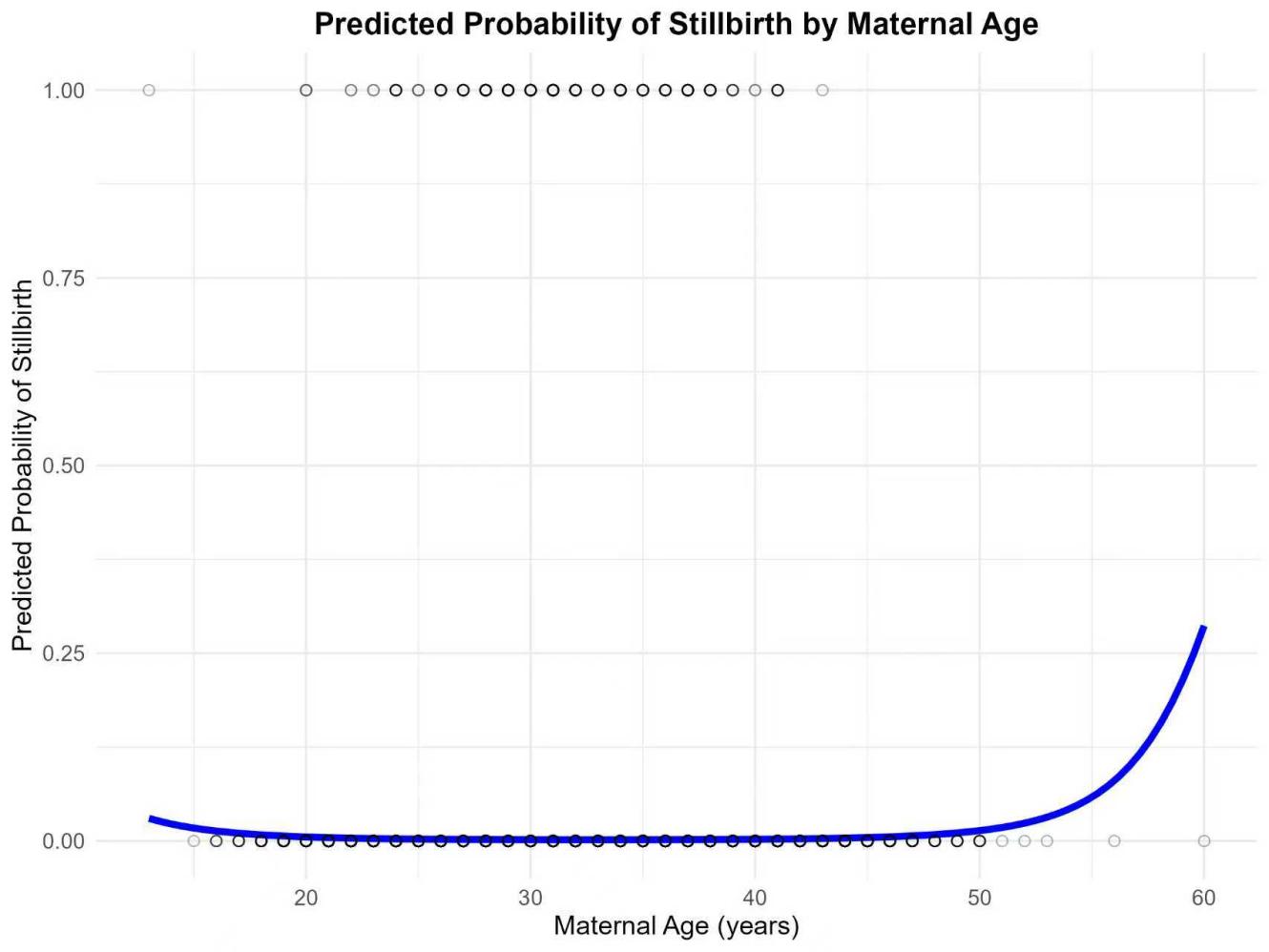


**Fig. S2 Predicted probability curve of stillbirth risk by maternal age**.

Using maternal age as a continuous variable with an added quadratic term in logistic regression, this plot illustrates the nonlinear U-shaped relationship between maternal age and stillbirth risk; the trough of the curve corresponds to 32.9 years, and young women (≤20 years) showed a more pronounced increase in risk than advanced-age women (40 years).
